# Social isolation and intrinsic capacity among older Hispanic adults in the United States: Exploring associations across geographic and social contexts

**DOI:** 10.1371/journal.pone.0357275

**Published:** 2026-09-02

**Authors:** Cai Xu, Juehyun Shin, Graciela Muniz-Terrera

**Affiliations:** Department of Social Medicine, Heritage College of Osteopathic Medicine, Ohio University, Athens, Ohio, United States of America; Virginia Commonwealth University School of Medicine, UNITED STATES OF AMERICA

## Abstract

**Objectives:**

We examined the association between social isolation (SI) and intrinsic capacity (IC) among older Hispanic adults in the United States and explored patterns of association across geographic and social subgroups.

**Methods:**

Using 2022 Health and Retirement Study data, we analyzed 615 Hispanic adults aged ≥60 years. IC (0–10) was derived from five domains, and SI (0–4) was measured using a composite index. Survey-weighted linear regression models were used to estimate the overall association between SI and IC. Exploratory interaction models and subgroup-specific analyses examined patterns across region of residence, community of residence, and nativity status.

**Results:**

Mean IC was 6.48 (SD = 1.68), and mean SI was 0.98 (SD = 0.91). In the overall sample, SI was not significantly associated with IC (β = −0.02, *p* > .05). Exploratory subgroup analyses identified several patterns. Among rural participants, higher SI was associated with lower IC (β = −0.63, *p* < .001), while among U.S.-born participants, higher SI was associated with lower IC (β = −0.29, *p* < .05). Across subgroups, higher household income and lower chronic disease burden were generally associated with higher IC(all *p* < .05).

**Conclusion:**

SI was not significantly associated with IC in the overall sample. Exploratory analyses identified potentially important patterns across residential and nativity groups, but these findings should be interpreted cautiously. Larger longitudinal studies are needed to clarify contextual differences and examine the pathways linking SI with functional aging among Hispanic older adults.

## Introduction

Social isolation (SI) has increasingly been recognized as a major public health concern among aging populations. A global meta‑analysis estimates that one in four community‑dwelling older adults worldwide are socially isolated [1]. In the United States, nationally representative data similarly indicate that approximately 24% of adults aged 65 and older, about 7.7 million individuals, are socially isolated, including 4% who are severely isolated [2]. Accumulating evidence links SI in later life to a range of adverse health outcomes [3], including increased risks of premature mortality [4], cardiovascular disease [5], depression [6], and cognitive decline [7]. As the U.S. population continues to age, identifying groups at heightened vulnerability and understanding contextual variation in SI have become critical public health priorities.

Importantly, SI is not evenly distributed across populations and may vary by social and geographic context. Data from the National Social Life, Health, and Aging Project indicate that rural older adults overall appear less socially isolated than their urban counterparts; however, this apparent rural advantage does not extend uniformly across racial and ethnic groups [8]. Findings from the National Health and Aging Trends Study further show that being unmarried, male, and having lower educational attainment and income are associated with a higher likelihood of SI [2]. In addition, reviews focusing on immigrant and racial/ethnic minority older adults suggest that ethnic identity and migration experiences intersect with conventional risk factors, contributing to heterogeneous patterns of SI and loneliness [9]. From a social determinant of health perspective, upstream structural conditions, including regional policy environments, health care access, neighborhood resources, and ethnic community density, may contribute to differences in both SI experiences and functional health outcomes, consistent with conceptualizations of social determinants as “causes of the causes” of health inequities [10]. Therefore, the association between SI and functional health may vary across geographic and social contexts.

Hispanic older adults represent one of the fastest‑growing aging populations in the United States. The Hispanic population aged 65 and older is projected to increase from just over 7% in 2012 to more than 18% by 2050 [11]. Emerging research shows that their experiences of SI and loneliness are highly heterogeneous across contexts. Although strong cultural traditions of familism and co-residence can buffer SI for some Hispanic/Latinx elders, SI and loneliness remain prevalent and are consistently linked to poorer mental health and, in many cases, worse physical health and comorbidity [12]. Structural disadvantages, including low income and education [12], multimorbidity [13], and migration-related factors and differences in access to healthcare and social resources across nativity groups [14], may contribute to variation in isolation risk among Hispanic older adults. Moreover, Hispanic older adults are disproportionately affected by chronic conditions and socioeconomic disadvantage [13], and are more likely to follow aging trajectories characterized by multimorbidity, functional limitation, and depressive symptoms [15], making multidimensional functional reserve particularly salient for understanding healthy aging trajectories in this population.

Intrinsic capacity (IC), proposed by the World Health Organization (WHO) as a framework for healthy aging, captures an individual’s composite physical and mental capacities [16]. Unlike disease-based measures, IC emphasizes overall functional reserves and resilience, making it particularly relevant for public health monitoring and prevention strategies [17]. Numerous longitudinal studies and systematic reviews indicate that lower IC and faster declines over time are strongly associated with a higher subsequent risk of multiple adverse health outcomes, including disability, falls, hospitalization, frailty, and mortality in middle‑aged and older adults [18]. Preserving higher IC trajectories is consistently linked to maintaining functional independence and delaying care dependency [19]. Research shows that SI is linked to lower IC [20], faster decline in its domains (especially cognition and psychological health) [7], and higher risk of later disability and frailty [21]. Understanding how SI relates to IC may help to identify early functional vulnerability, before disability or other adverse outcomes occur.

Despite growing recognition of SI as an important determinant of health among Hispanics in the United States, existing research on older Hispanic/Latino adults has largely focused on its association with single health outcomes, such as cognitive, psychological, or physical functioning [12,22,23]. Few studies have examined the relationship between SI and multidimensional functional reserve within the WHO IC framework among U.S. Hispanic/Latino older adults [20]. In addition, limited evidence exists regarding whether the association between SI and IC varies across different segments of the Hispanic older adult population, including those defined by nativity status, residential community, and geographic region. Despite well-documented regional, nativity, and sociodemographic heterogeneity in health outcomes within the U.S. Hispanic population [24,25], the association between SI and IC across these contexts remains insufficiently characterized.

Therefore, this study examined the association between SI and IC among older Hispanic adults in the United States. The primary objective was to evaluate the overall association between SI and IC in a nationally representative sample. Given potential heterogeneity in social and geographic contexts, we additionally conducted exploratory analyses to examine whether the estimated SI–IC association varied across region of residence, community of residence, and nativity status. Specifically, we tested SI × region of residence, SI × community of residence, and SI × place of birth interaction terms in the full sample. We also conducted subgroup-specific analyses stratified by region of residence (Northeast-Midwest, South, and West), community of residence (rural vs. urban), and nativity status (foreign-born vs. U.S.-born) to characterize associations between SI and IC within these groups. These analyses were intended to describe potential patterns of heterogeneity and generate hypotheses for future research rather than to establish definitive effect modification. By examining SI–IC associations across diverse Hispanic subgroups, this study provides preliminary evidence regarding potential heterogeneity in the relationship between SI and IC among a heterogeneous aging population. Findings may inform future research on the mechanisms and longitudinal pathways linking SI with functional aging.

## Methods

### Data source

We used data from the publicly available Health and Retirement Study (HRS), a nationally representative longitudinal survey of U.S. adults aged 50 and older. Since its launch in 1992, the HRS has collected rich information on health, cognition, economic status, work and retirement, and family connections, surveying over 37,000 individuals across more than 23,000 households [26]. The study oversamples Black and Hispanic populations, enabling us to examine diverse subgroups and making it a uniquely valuable resource for aging research. For our analysis, we focused on the latest available 2022 wave, which initially included 15,856 participants. We excluded individuals younger than 60, those identifying as non-Hispanic or with unknown ethnicity, and participants with substantial missing data on key variables such as vitality index and and survey design variables, resulting in a final analytic sample of 615 participants (see Appendix Fig 1 in S1 Fig). The HRS protocol was approved by the University of Michigan Institutional Review Board (IRB Protocol No. HUM00061128), and all participants provided written informed consent prior to participation. As this study used publicly available, de-identified HRS data, additional institutional review board approval was not required.

### Outcome

IC was the outcome variable and was calculated by summing scores across five domains: cognition, psychological ability, sensory function, locomotion, and vitality [27]. Specifically, cognition included memory and orientation; psychological ability was assessed via depressive symptoms; sensory function encompassed vision and hearing; locomotion measured walking and chair-stand difficulties; and vitality was evaluated using grip strength and BMI. Each domain was scored 0–2 based on standardized criteria, yielding a total IC score ranging from 0 to 10, with higher scores indicating better overall functional capacity (See Appendix Tables 1 and 2 in S2 Table for details of IC Score generation).

### Major predictor

SI, the primary predictor in this study, was operationalized as structural social isolation using an established HRS-based composite measure [28]. The score ranged from 0 (socially integrated) to 4 (socially isolated). It reflects both the presence of social ties, including a living partner, children, other family members, and friends, and the frequency of contact with these ties. Each component was scored 0 or 1 based on whether contact was frequent or infrequent, and the total score was calculated by summing across components, with higher scores indicating greater SI. This measure captures the extent of an individual’s social engagement and support network, which has been linked to functional and cognitive outcomes in older adults (see Appendix Tables 1 and 3 in S2 Table).

### Covariates

Several demographic, socioeconomic, and health-related variables were included as covariates in the statistical models (see Appendix Table 1 in S2 Table). Age was included as a continuous variable (years). Sex was categorized as female (reference) or male, and race was categorized as White/Caucasian or non-White (reference). Educational attainment was classified as primary (reference), secondary, or tertiary education. Marital status was categorized as single/divorced (reference) or married/partnered. Household income was categorized into tertiles (low [reference], middle, and high). Community of residence was classified as rural or urban based on the United States Department of Agriculture (USDA) Beale codes, with rural residence as the reference group. Region of residence was based on participants’ current residential location and categorized as West, South, or Northeast-Midwest (reference); the Northeast and Midwest regions were combined due to limited sample sizes in individual regions. Place of birth was categorized as foreign-born (reference) or U.S.-born. Health-related covariates included the number of chronic conditions, defined as the presence of hypertension, diabetes, stroke, cardiovascular disease, lung disease, or cancer, and categorized as none (reference), one, or two or more conditions. Physical activity was categorized as physically inactive (reference) or physically active based on the HRS items assessing participation in vigorous, moderate, or mild physical activities. Participants reporting any such activity more than once per week or every day were classified as physically active; all others were classified as physically inactive [27]. Alcohol consumption was categorized by drinking frequency as no drinking (reference; 0 days/week or never), low-to-moderate frequency drinking (1–3 days/week), or higher-frequency drinking (≥4 days/week).

### Analytical strategies

The proportion of missing data ranged from 0.16% for marital status and community of residence, 2.11% for race, to 33.50% for SI. To evaluate the missing-data mechanism, we modeled the probability of missing SI using logistic regression, with demographic, socioeconomic, health-related, geographic, and IC variables as predictors. Missingness in SI was significantly associated with age, nativity, and educational attainment (*p* < .05), but not with IC (*p* = .31). These observed missingness patterns were considered compatible with the missing-at-random (MAR) assumption, although MNAR could not be ruled out.

Before model fitting, multicollinearity among covariates was assessed. All analyses incorporated the HRS 2022 respondent-level analysis weight (R16WTRESP) and accounted for the complex survey design using sampling strata (STRATUM) and primary sampling units (SECU). Missing data were handled using multiple imputation by chained equations (MICE) with 30 imputations [29]. The imputation model included IC, SI, and all prespecified covariates. Survey design objects incorporating sampling weights, strata, and primary sampling units were specified for each imputed dataset, and survey-weighted linear regression models were fitted within each dataset. Estimates were combined using Rubin’s rules. Analyses were conducted in the overall sample using a model without interaction terms as the primary analysis, followed by exploratory models including SI × region of residence, SI × community of residence, and SI × place of birth interaction terms. Exploratory subgroup-specific analyses were subsequently conducted by region of residence, community of residence, and place of birth. All models were adjusted for the prespecified covariates. Regression coefficients (β) and standard errors (SE) are reported, and statistical significance was defined as a two-sided p < 0.05. All analyses were performed using R version 4.5.1.

## Results

### Sample characteristics

**Fig 1:**
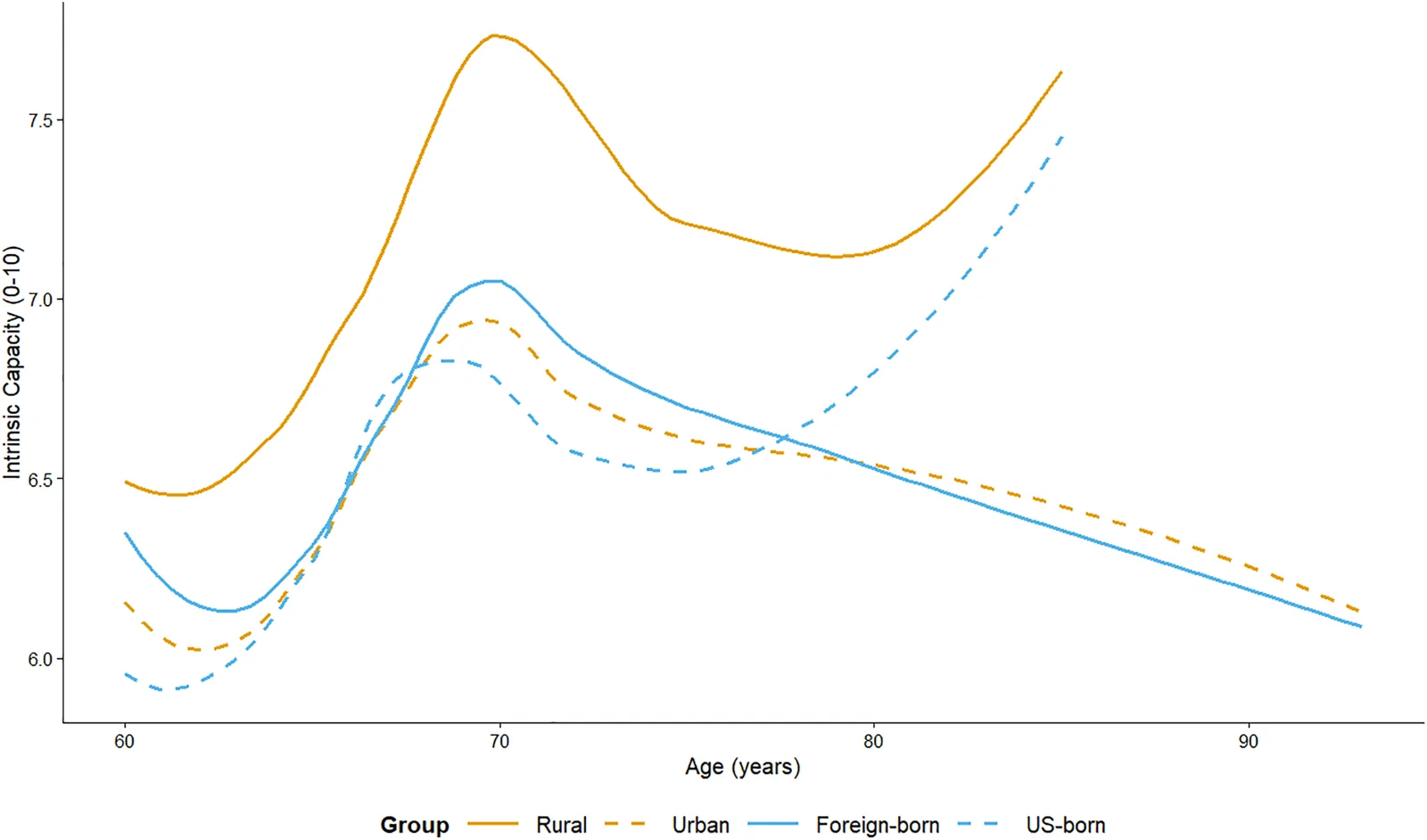
Intrinsic capacity by age according to residence and birthplace before multiple imputation. *Note:* The figure was generated using observed (pre-imputation) data. Participants with missing information on variables required for the visualization were excluded, resulting in an analytic sample of 614 participants.

Table 1 presents the baseline characteristics of the 615 participants before multiple imputation. The mean age of the study population was 68.92 years (SD = 7.04), 59.35% were female, and the mean IC score was 6.48 (SD = 1.68). Overall, 64.23% of participants were foreign-born, 92.03% resided in urban communities, and 54.31% reported two or more chronic conditions.Compared with urban participants, rural participants were more likely to have tertiary education, reside in the West, be U.S.-born, and report higher IC scores (all *p* < .05). Compared with U.S.-born participants, foreign-born participants were more likely to have primary education, lower household income, live in urban areas, and report no alcohol consumption (all *p* < .05). Participants in the Northeast/Midwest were more likely to be non-White, unmarried/divorced, foreign-born, and have lower household income compared with those in other regions(all *p* < .05)(See Appendix Table 4 in S2 Table). As shown in Fig 1, predicted IC trajectories demonstrated nonlinear age-related patterns. Rural participants generally showed higher predicted IC levels than urban participants. Differences by nativity varied across age, with foreign-born participants demonstrating higher IC levels at younger ages and less advantage at older ages.

**Table 1:** Descriptive statistics of the 2022 HRS study sample (n = 615) before multiple imputation.

Characteristics	Code	Category	Overall
Age—mean(SD)		Numerical	68.92(7.04)
Gender—n(%)			
	0	Female	365(59.35)
	1	Male	250(40.65)
Race—n(%)			
	0	Non-white	271(44.07)
	1	White/Caucasian	331(53.82)
		Unknown	13 (2.11)
Educational levels—n(%)			
	0	Primary	270(43.9)
	1	Secondary	260(42.28)
	2	Tertiary	85(13.82)
Marital status—n(%)			
	0	Single/Divorced	227(36.91)
	1	Married/Partnered	387(62.93)
		Unknown	1(0.16)
Household income —n(%)			
	0	≤ $17,204	205(33.33)
	1	$17,204–$40,810.67	205(33.33)
	2	>$40,810.67	205(33.33)
Community of residence —n(%)			
	0	Rural	48(7.8)
	1	Urban	566(92.03)
		Unknown	1(0.16)
Region of residence—n(%)			
	0	Northeast-Midwest	92(14.96)
	1	South	242(39.35)
	2	West	281(45.69)
Place of birth—n(%)			
	0	Foreign-born	395(64.23)
	1	US-born	220(35.77)
Number of chronic diseases—n(%)			
	0	None	105(17.07)
	1	1	176(28.62)
	2	2+	334(54.31)
Physical activity—n(%)			
	0	Physically inactive	211(34.31)
	1	Physically active	404(65.69)
Alcohol consumption—n(%)			
	0	No drinking	413(67.15)
	1	Low-to-moderate frequency	167(27.15)
	2	Higher-frequency drinking	35(5.69)
Social isolation—mean(SD)		Numerical	0.98(0.91)
		Unknown	206(33.50)
Intrinsic capacity—mean(SD)		Numerical	6.48(1.68)

*Notes*: Continuous variables are presented as mean (SD) and categorical variables as n (%), based on observed (pre-imputation) data.

Compared with excluded participants, those included in the analytic sample were younger (mean age: 68.92 vs. 70.27 years, *p* < .001), more likely to reside in urban communities (92.03% vs. 84.73%, *p* < .001), had lower SI scores (mean: 0.98 vs. 1.37, *p* = .002), and had higher IC scores (mean: 6.48 vs. 5.38, *p* < .001) (Appendix Table 5 in S2 Table). Multicollinearity checks (Appendix Table 6 in S2 Table) revealed no concerns for the IC model.

### Association between SI and IC

In fully adjusted models after multiple imputation (see Appendix Table 7 in S2 Table), SI was not significantly associated with IC (β = −0.02, *p* > .05). No significant interactions were observed between SI and region of residence or place of birth. In the interaction model, the SI coefficient was statistically significant for the reference group (rural participants) (β = −0.39, *p* < .05), while the SI × community of residence interaction was not statistically significant (β = 0.40, SE = 0.23). Higher household income was consistently associated with higher IC across models (β = 0.57–0.69, *p* < .05), whereas having ≥2 chronic diseases was associated with lower IC (β = −1.13, *p* < .001).

In residence-specific analyses (Table 2), higher SI was significantly associated with lower IC among rural participants (β = −0.63, *p* < .001), as was having ≥2 chronic diseases (β = −0.93, *p* < 0.01). Among urban participants, higher household income was associated with higher IC (β = 0.57–0.75, *p* < .05), whereas having ≥2 chronic diseases was associated with lower IC (β = −1.19, *p* < .001).

**Table 2:** Subgroup-specific associations between social isolation and intrinsic capacity by residential community(n = 615).

	Community of residence	
	Rural	Urban
	β(SE)	β(SE)
Age	0.06**(0.02)	0.05***(0.01)
Social isolation	−0.63***(0.19)	0.01 (0.14)
Region of residence		
Northeast-Midwest(ref.)		
South	−0.76 (0.95)	−0.11 (0.27)
West	0.18 (0.76)	−0.26 (0.25)
Gender		
Female(ref.)		
Male	0.18 (0.49)	−0.08 (0.24)
Race		
Non-white(ref.)		
White/Caucasian	0.37 (0.39)	−0.09 (0.20)
Educational levels		
Primary (ref.)		
Secondary	−0.21 (0.57)	0.09 (0.22)
Tertiary	0.66 (0.83)	0.59 (0.47)
Marital status		
Single/Divorced(ref.)		
Married/Partnered	0.50 (0.33)	0.32 (0.23)
Household income		
≤ $17,204(ref.)		
$17,204–$40,810.67	−0.08 (0.44)	0.57*(0.26)
> $40,810.67	−0.54 (0.47)	0.75**(0.26)
Place of birth		
Foreign-born(ref.)		
US-born	−0.28 (0.53)	−0.05 (0.20)
Number of chronic diseases		
None(ref.)		
1	−0.68 (0.42)	−0.19 (0.23)
2+	−0.93**(0.35)	−1.19***(0.22)
Physical activity		
Physically inactive(ref.)		
Physically active	0.58 (0.50)	0.18 (0.24)
Alcohol consumption		
No drinking (ref.)		
Low-to-moderate frequency	−0.12 (0.33)	0.22 (0.16)
Higher-frequency drinking	1.74 (1.06)	0.11 (0.51)

*Notes*: Estimates were obtained from survey-weighted linear regression models incorporating HRS sampling weights and pooled across 30 multiply imputed datasets using Rubin’s rules. Values are presented as β (SE). Residence-based subgroup estimates should be interpreted with caution given the small rural sample size. Interaction analyses involving social isolation and region were not conducted within residence subgroups because of limited sample size and concerns regarding model stability. *p < .05; **p < .01; ***p < .001.

In region-specific analyses (Table 3), higher educational attainment was associated with higher IC in the Northeast-Midwest (β = 0.84–1.84, *p* < .05) and South (β = 1.23, *p* < .05). Higher household income was also associated with higher IC in the Northeast-Midwest (β = 1.06–1.49, *p* < .001). Having ≥2 chronic diseases was associated with lower IC across all regions (β = −0.82 to −1.40, *p* < .05), while physical activity was positively associated with IC in the West (β = 0.90, *p* < .001).

**Table 3:** Subgroup-specific associations between social isolation and intrinsic capacity by region of residence (n = 615).

	Region of residence		
	Northeast-Midwest	South	West
	β(SE)	β(SE)	β(SE)
Age	−0.02*(0.01)	0.05**(0.02)	0.07***(0.01)
Social isolation	−0.26 (0.16)	−0.08 (0.19)	0.12 (0.20)
Gender			
Female(ref.)			
Male	0.05 (0.33)	−0.06 (0.19)	0.06 (0.30)
Race			
Non-white(ref.)			
White/Caucasian	0.35 (0.31)	−0.09 (0.27)	−0.21 (0.25)
Educational levels			
Primary (ref.)			
Secondary	0.84*(0.38)	0.27 (0.32)	−0.33 (0.28)
Tertiary	1.84***(0.53)	1.23*(0.49)	0.14 (0.45)
Marital status			
Single/Divorced (ref.)			
Married/Partnered	0.16 (0.28)	0.65 (0.42)	0.29 (0.27)
Household income			
≤ $17,204(ref.)			
$17,204–$40,810.67	1.49***(0.39)	0.10 (0.51)	0.32 (0.33)
> $40,810.67	1.06***(0.25)	0.60 (0.38)	0.53 (0.43)
Place of birth			
Foreign-born(ref.)			
US-born	0.11 (0.39)	−0.32 (0.17)	0.15 (0.37)
Community of residence			
Rural (ref.)			
Urban	1.26*(0.53)	−0.31 (0.43)	−0.33 (0.32)
Number of chronic diseases			
None (ref.)			
1	0.08 (0.37)	−0.21 (0.24)	−0.61**(0.23)
2+	−0.82*(0.33)	−0.84***(0.24)	−1.40***(0.19)
Physical activity			
Physically inactive (ref.)			
Physically active	−0.46 (0.33)	−0.40 (0.28)	0.90***(0.22)
Alcohol consumption			
No drinking(ref.)			
Low-to-moderate frequency	−0.30 (0.28)	0.44*(0.19)	0.26 (0.22)
Higher-frequency drinking	0.14 (0.50)	−0.64 (0.73)	0.65 (0.42)

*Notes:* Results are based on 30 multiply imputed datasets and pooled using Rubin’s rules. Estimates were obtained from survey-weighted linear regression models. Values are presented as β (SE). *p < .05; **p < .01; ***p < .001.

In nativity-specific analyses (Table 4), higher SI was associated with lower IC among U.S.-born participants (β = −0.29, *p* < .05). Among foreign-born participants, higher household income was associated with higher IC (β = 0.63–1.03, *p* < .05), whereas having ≥2 chronic diseases was associated with lower IC (β = −1.04, *p* < .001). Among U.S.-born participants, tertiary education was associated with higher IC (β = 1.31, *p* < .001), while having one or ≥2 chronic diseases was associated with lower IC (β = −1.03 and −1.52, respectively, *p* < .001).

**Table 4:** Subgroup-specific associations between social isolation and intrinsic capacity by nativity status(n = 615).

	Place of birth	
	Foreign-born	US-born
	β(SE)	β(SE)
Age	0.03*(0.01)	0.08***(0.02)
Social isolation	0.04 (0.18)	−0.29*(0.14)
Region of residence		
Northeast-Midwest(ref.)		
South	0.11 (0.29)	−0.41 (0.47)
West	−0.21 (0.27)	−0.30 (0.45)
Gender		
Female(ref.)		
Male	−0.43 (0.38)	0.54 (0.30)
Race		
Non-white(ref.)		
White/Caucasian	−0.20 (0.21)	0.38 (0.31)
Educational levels		
Primary (ref.)		
Secondary	0.06 (0.26)	0.38 (0.23)
Tertiary	0.28 (0.55)	1.31***(0.34)
Marital status		
Single/Divorced (ref.)		
Married/Partnered	0.59*(0.27)	−0.19 (0.26)
Household income		
≤ $17,204(ref.)		
$17,204–$40,810.67	0.63*(0.27)	0.24 (0.26)
> $40,810.67	1.03**(0.35)	−0.01 (0.28)
Community of residence		
Rural (ref.)		
Urban	−0.38 (0.39)	0.02 (0.25)
Number of chronic diseases		
None (ref.)		
1	0.04 (0.28)	−1.03***(0.31)
2+	−1.04***(0.27)	−1.52***(0.27)
Physical activity		
Physically inactive (ref.)		
Physically active	−0.08 (0.26)	0.54 (0.29)
Alcohol consumption		
No drinking (ref.)		
Low-to-moderate frequency	0.23 (0.25)	0.29 (0.24)
Higher-frequency drinking	−0.42 (0.64)	0.51 (0.38)

*Notes*: Results are based on 30 multiply imputed datasets and pooled using Rubin’s rules. Estimates were obtained from survey-weighted linear regression models. Values are presented as β (SE).

* *p* < .05; **p < .01; ***p < .001.

## Discussion

This study examined the association between SI and IC among Hispanic adults aged 60 and older in the 2022 HRS. In the overall sample, SI was not significantly associated with IC. Exploratory subgroup analyses identified negative associations between SI and IC among rural and U.S.-born participants, although these subgroup-specific findings should be interpreted cautiously because the corresponding interaction tests did not provide sufficient evidence to establish statistically significant effect modification. These findings add to a growing literature positioning SI as a potentially modifiable determinant of later-life health [30]. Using nationally representative samples from the United States (HRS) and Mexico (Mexican Health and Aging Study [MHAS]), Wilson and Marini (2024) demonstrated that older adults cluster into distinct social profiles defined by loneliness, living arrangement, and relationship support, which are differentially associated with functional and inflammatory outcomes [31]. Further, our results align with work highlighting the importance of place-based structural conditions for later-life functioning. Lamar et al. (2023) reported that higher neighborhood social vulnerability was associated with worse cognitive and motor functioning in older non-Latino Black and Latino adults, and that domains most strongly linked to neighborhood vulnerability varied by ethno-racial group [32].

Prior studies conducted primarily among non-Hispanic White populations have reported associations between SI and functional decline [33–36], whereas we did not observe a statistically significant association between SI and IC in our sample. Several factors may contribute to differences in findings across studies, although direct comparisons are limited because of differences in populations, measures, and study designs. First, we used a multidomain IC framework integrating cognition, psychological capacity, sensory function, locomotion, and vitality rather than traditional activities of daily living/ instrumental activities of daily living (ADL/IADL) outcomes [36]. As IC captures multiple dimensions of functional reserve simultaneously, an association between SI and one specific functional domain may not necessarily translate into a detectable association with the overall IC construct. Second, loneliness and SI are related but distinct constructs. In MHAS, loneliness was assessed using a brief UCLA loneliness scale, whereas SI was assessed using a social network index incorporating partnership status, religious activity, group membership, and closeness ties [37]. These differences in measurement may partly contribute to the differing findings across studies.

Consistent with this interpretation, prior studies emphasize within-group heterogeneity in psychosocial correlates of health among Hispanics/Latinos. Estrella et al. (2021) reported that social support and purpose in life were positively associated with cognitive performance, while loneliness was negatively associated with multiple domains; they also found that culturally relevant factors (e.g., ethnic identity, familism) were not uniformly protective [38]. Miyawaki (2015) similarly reported that associations between SI and health varied in strength and direction across racial/ethnic groups and across isolation dimensions (perceived vs. disconnectedness), with consistent links to self-rated mental health among Hispanic older adults [23].

In residence-specific analyses, higher SI was associated with lower IC among rural participants, whereas this association was not observed among urban participants. However, the interaction between SI and community of residence was not statistically significant, indicating that the observed rural-specific association should not be interpreted as evidence of a definitive rural–urban difference in the SI–IC association.Several structural and contextual factors may contribute to this pattern, including differences in transportation availability, healthcare access, financial resources, language accessibility, and access to culturally appropriate services. However, these factors were not directly measured in the present study and should therefore be considered potential explanations rather than established mechanisms [39]. Further research is needed to examine whether and how rural contexts shape the relationship between SI and IC.

Nativity-specific analyses showed a significant negative association between SI and IC among U.S.-born participants, but not among foreign-born participants. This pattern should be interpreted cautiously because the corresponding interaction analysis did not provide evidence of statistically significant effect modification. Nevertheless, the observed variation across nativity groups is consistent with evidence that Hispanic/Latino health outcomes may differ by nativity and other sociodemographic characteristics, suggesting that nativity may be an important context for understanding heterogeneity in late-life health [40]. Differences in migration-related experiences, acculturation, social networks, and access to social resources may potentially contribute to such variation, although these mechanisms were not directly assessed in the present study [41].

Of note, higher educational attainment and household income were generally associated with higher IC, whereas having two or more chronic diseases was consistently associated with lower IC. These patterns are consistent with previous research identifying education, socioeconomic position, and chronic disease burden as important correlates of IC. A longitudinal study of older adults in Mexico found that higher education and wealth were associated with more favorable IC trajectories [42], while a recent scoping review similarly identified education, income/wealth, and chronic diseases among the factors consistently associated with IC [43]. These findings are consistent with the multidimensional nature of IC, which reflects both accumulated health burden and socioeconomic resources.

### Implications for policy and practice

Our findings suggest that efforts to support healthy aging and improve IC among older Hispanic adults may benefit from considering social and geographic contexts rather than assuming uniform patterns across populations. Although subgroup analyses suggested potential variation across residential and nativity contexts, these findings should be interpreted cautiously given limited subgroup power and the lack of statistically significant interaction effects. Thus, the present findings should be viewed as a rationale for further evaluation rather than as evidence supporting specific context-tailored interventions. Future intervention research could examine whether strategies that promote social participation and meaningful social connections are effective in supporting IC, particularly when integrated with broader multicomponent approaches to healthy aging that address multiple domains of IC [30]. Programs integrating physical activity, nutrition, cognitive training, and psychosocial support, along with person-centered goal setting, may provide potential frameworks for future intervention development and evaluation, rather than established recommendations based on the present study [44]. Notably, few existing interventions explicitly use IC as a guiding framework, highlighting an opportunity for future program design and evaluation [44]. Finally, future research should examine how regional, cultural, and linguistic contexts may influence the effectiveness of interventions addressing social disconnection among diverse Hispanic older adults. Culturally responsive approaches, including attention to language accessibility and community-specific needs, could be explored in future studies to determine whether they can improve social connectedness and functional health [39]. More broadly, future research could also examine whether incorporating IC assessment into population health initiatives may help identify older adults with lower functional capacity, particularly when considered alongside relevant social and contextual factors [45].

### Limitations

This study has several limitations. *First*, the cross-sectional design precludes establishing temporality, and reverse causation is possible if declining IC reduces social engagement. *Second*, SI was assessed as structural social isolation using an established HRS-based index that captures the availability and frequency of social ties [28]. However, it does not capture subjective loneliness, relationship quality, or negative social interactions. Future studies using multidimensional measures of social relationships may provide a more comprehensive assessment of social isolation. *Third,* although multiple imputation was used under a missing-at-random assumption, estimates may still be biased if missingness was related to unobserved factors associated with SI or IC. In addition, the final analytic sample was smaller than the eligible Hispanic population because of missing IC components, partly related to the HRS rotating half-sample design for physical measures. We compared included and excluded participants and incorporated survey weights to address potential differences; however, residual selection bias cannot be completely excluded. *Fourth*, subgroup analyses, particularly among rural participants (n = 48), had limited statistical power. Although these analyses identified potentially meaningful patterns, the small subgroup sizes may have resulted in imprecise estimates and limited the ability to detect meaningful differences between groups. Accordingly, subgroup-specific findings should be interpreted as exploratory and warrant confirmation in larger samples. *Fifth*, residual confounding from unmeasured factors, including neighborhood characteristics, migration history, and other social or structural conditions, cannot be excluded. *Sixth*, the HRS provides limited information on country of origin and Hispanic subgroup identity, which limited our ability to examine heterogeneity within the Hispanic population. Future studies with more detailed subgroup measures are needed to further explore within-group differences. *Finally*, the vitality domain was assessed using handgrip strength and BMI, consistent with previous HRS-based studies and commonly used approaches in the intrinsic capacity literature [27,46]. However, BMI does not differentiate fat mass from lean muscle mass and may not fully capture conditions such as sarcopenic obesity. Future studies incorporating direct measures of body composition or nutritional status may provide a more comprehensive assessment of vitality. In addition, the use of a composite IC score may mask domain-specific associations between SI and individual IC domains.

## Conclusion

In this nationally representative sample of older Hispanic adults, SI was not significantly associated with IC in pooled analyses. Exploratory subgroup analyses identified negative associations between SI and IC among rural and U.S.-born participants, although these patterns were not supported by statistically significant interaction tests. These findings suggest that the overall association between SI and multidimensional functional capacity was limited in this sample, while subgroup-specific patterns warrant further investigation. Adequately powered longitudinal studies are needed to clarify the temporal and contextual relationships between SI and IC.

## Supporting information

S1 Fig Flowchart for participants from the Health and Retirement Study (HRS) 2022 wave. (DOCX)

S2 Table Appendix Tables 1–8. (DOCX)
