# Supplementary material for: Social isolation and intrinsic capacity among older Hispanic adults in the United States: Exploring associations across geographic and social contexts
Source: PLoS One. 2026 Sep 2;21(9):e0357275. doi: 10.1371/journal.pone.0357275 (PMC13537567; doi:10.1371/journal.pone.0357275)
Supplement: Supplementary file 1 [file pone.0357275.s001.docx]

**S1 Fig 1. Flowchart for participants from the Health and Retirement Study (HRS) 2022 wave.**

Total participants in the HRS 2022 wave

(N=15,856)

C

Excluded participants aged < 60 years

(n = 3,942)

Participants aged 60 years and older

(n = 11,914)

Excluded non-Hispanics or unknown ethnicity

(n = 9,819)

Hispanics participants

(n = 2,095)

Excluded participants with missing data on the vitality index and survey design variables

(n = 1,480)

Hispanic participants aged≥60 years included in the final analysis

(n=615)

Note: Missingness in some IC components, particularly vitality measures, was partly attributable to the HRS rotating half-sample design for physical measures. Participants with missing required IC or survey design variables were therefore excluded from the final analytic sample.
