# Supplementary material for: Social isolation and intrinsic capacity among older Hispanic adults in the United States: Exploring associations across geographic and social contexts
Source: PLoS One. 2026 Sep 2;21(9):e0357275. doi: 10.1371/journal.pone.0357275 (PMC13537567; doi:10.1371/journal.pone.0357275)
Supplement: Supplementary file 2 [file pone.0357275.s002.docx]

**S2 Table 1. Brief introduction of included variables for this current study.**

| **Variable** | **Classification** | **Brief description** |
| --- | --- | --- |
| Age | Numerical | Interviewee’s validated age |
| Gender | Categorical | Gender as reported by the interviewee |
|  | (0) Female [Reference] |  |
|  | (1) Male |  |
| Race | Categorical | Race as reported by the interviewee |
|  | (0) Non-white [Reference] |  |
|  | (1) White/Caucasian |  |
| Educational levels | Categorical | Highest degree received as reported by the interviewee |
|  | (0) Primary [Reference] |  |
|  | (1) Secondary |  |
|  | (2) Tertiary |  |
| Marital status | Categorical | Current marital status as reported by the interviewee |
|  | (0) Single/Divorced [Reference] |  |
|  | (1) Married/Partnered |  |
| Household income | Categorical | Total household income from respondent and spouse, classified into tertiles (1/3 each) based on the distribution: Tertile 1 (lowest), Tertile 2 (middle), Tertile 3 (highest). |
|  | (0) ≤ $17,204 [Reference] |  |
|  | (1) $17,204–$40,810.67 |  |
|  | (2) > $40,810.67 |  |
| Community of residence | Categorical | Current residential area:  Coded based on the 2022 Beale Rural-Urban Continuum codes. |
|  | (0) Rural [Reference] |  |
|  | (1) Urban |  |
| Region of residence | Categorical | Current residential region of the participant, with Midwest and Northeast combined into “Northeast-Midwest” group for geographic clarity. |
|  | (0) Northeast-Midwest [Reference] |  |
|  | (1) South |  |
|  | (2) West |  |
| Place of birth | Categorical | Place of birth based on reported regions by the interviewee |
|  | (0) Foreign-born [Reference] |  |
|  | (1) US-born |  |
| Number of chronic diseases | Categorical | Number of Chronic disease (asking if they have including hypertension, diabetes, stroke, cardiovascular diseases, lung diseases, and cancer), as reported by the interviewee |
|  | (0) None [Reference] |  |
|  | (1) 1 |  |
|  | (2) ≥ 2 |  |
| Physical activity | Categorical | Participants reporting engagement in vigorous, moderate, or mild physical activity more than once per week or daily were classified as physically active; all others were classified as physically inactive. |
|  | (0) Physically inactive [Reference] |  |
|  | (1) Physically active |  |
| Alcohol consumption | Categorical | Number of days per week the participant reported drinking alcohol, categorized as no drinking (0 days/week or never), low-to-moderate frequency drinking (1–3 days/week), or higher-frequency drinking (≥4 days/week). |
|  | (0) No drinking [Reference] |  |
|  | (1) Low-to-moderate frequency |  |
|  | (2) Higher-frequency drinking |  |
| Social isolation | Numerical | Social isolation was measured using a composite score derived from the presence of social ties (partner, children, family, friends) and frequency of contact with these ties. Scores ranged from 0 (socially integrated) to 4 (socially isolated) |
| Intrinsic capacity (IC) | Numerical | IC was assessed across five domains—cognition, psychological ability, sensory function, locomotion, and vitality (score range 0–10), with higher scores indicating better IC. Details are in S2 Table 2. |

*Note*: Physical activity was based on HRS items assessing (1) vigorous activities (e.g., running/jogging, swimming, cycling, aerobics, gym workouts, tennis, or digging with a spade/shovel), (2) moderately energetic activities (e.g., gardening, cleaning the car, moderate-paced walking, dancing, or floor/stretching exercises), and (3) mildly energetic activities (e.g., vacuuming, laundry, or home repairs). Response categories were: 1 = more than once a week; 2 = once a week; 3 = one to three times a month; 4 = hardly ever or never; 7 = every day/every day.

**S2 Table 2**. **Measurements of intrinsic capacity (IC) from HRS 2022 wave survey.**

| **Domains** | **Variables** | **Score** | **Criteria / Cut-off** | **Measurement Description** |
| --- | --- | --- | --- | --- |
| Cognition | Memory | 0 | ≤ (Mean - SD) | Immediate recall of 10 words and delayed recall of the same 10 words, with cut-off points stratified by age and gender groups^1^. |
|  |  | 1 | > (Mean - SD) |  |
|  | Orientation | 0 | ≤ (Mean - SD) | Assessed by recalling the year, month, day, and day of the week, with cut-off points stratified by age and gender groups. |
|  |  | 1 | > (Mean - SD) |  |
| Psychological ability | Depression | 0 | 4–8 | Center for Epidemiologic Studies Depression Scale; CES-D (8 items)^2^  Assessed using eight items from the CES-D scale, including feelings of depression, effort, restless sleep, happiness (reversed), loneliness, enjoyment of life (reversed), sadness, and lack of motivation during the past week. Each item was coded as yes/no, and the total score ranged from 0 to 8, with higher scores indicating more depressive symptoms. |
|  |  | 1 | 2-3 |  |
|  |  | 2 | 0-1 |  |
| Sensory function | Vision | 0 | Poor | Self-rated vision  eyesight using glasses or corrective lenses as needed |
|  |  | 1 | Excellent, very good, good, or fair |  |
|  | Hearing | 0 | Poor | Self-rated hearing  using a hearing aid as usual |
|  |  | 1 | Excellent, very good, good, or fair |  |
| Locomotion | Walking difficulty | 0 | Yes | Self-rated difficulty with walking one block |
|  |  | 1 | No |  |
|  | Chair stand difficulty | 0 | Yes | Self-rated difficulty with getting up from a chair after sitting for long periods |
|  |  | 1 | No |  |
| Vitality | Grip strength | 0 | Men: < 30 kg;  Women: < 20 kg | Handgrip strength was measured using a dynamometer. Participants with at least one trial were included, and the maximum value from either the left or right hand was used for analysis^3^. |
|  |  | 1 | Men: ≥ 30 kg;  Women: ≥ 20 kg |  |
|  | BMI | 0 | Underweight: < 18.5  Obese: ≥ 30 | Calculated as weight (kg) divided by height squared (m²), using converted values from pounds and inches to kilograms and meters. Participants were classified into two groups based on gender-specific BMI cut-off points. |
|  |  | 1 | Normal weight:18.5–24.9  Overweight:25–29.9 |  |

1.Petersen RC, Roberts RO, Knopman DS, et al. Prevalence of mild cognitive impairment is higher in men: The Mayo Clinic Study of Aging. Neurology. 2010;75(10):889-897.

2.White J, Zaninotto P, Walters K, et al. Severity of depressive symptoms as a predictor of mortality: the English longitudinal study of ageing. Psychol Med. 2015;45(13):2771-2779.

3.Cruz-Jentoft AJ, Baeyens JP, Bauer JM, et al. Sarcopenia: European consensus on definition and diagnosis: Report of the European Working Group on Sarcopenia in Older People. Age Ageing. 2010;39(4):412-423.

**S2 Table 3. Measurements of social isolation (SI) from HRS 2022 wave survey.**

| **Domain** | **Question / Item** | **Response Options** | **Recording / Scoring** |
| --- | --- | --- | --- |
| Composition of Social Network^a^ | 1. Do you have a husband, wife, or partner with whom you live? | 1 = Yes, 0 = No | *Reverse coded*: 1 = No, 0 = Yes |
|  | 2. Do you have any living children? | 1 = Yes, 0 = No |  |
|  | 3. Do you have any other immediate family (e.g., brothers, sisters, parents, cousins, or grandchildren)? | 1 = Yes, 0 = No |  |
|  | 4. Do you have any friends? | 1 = Yes, 0 = No |  |
|  | *Total scoring rule* |  | 1 = No, 0 = Yes  Range:0-1 |
| Contact with Children (not living with you) | a. Meet up (arranged or chance meetings) | 1 = ≥3 times/week.  2 = 1–2 times/week;  3 = 1–2 times/month.  4 = every few months;  5 = 1–2 times/year;  6 = <1 time/year or never | Recoded: 1 = 4–6 (infrequent),  0 = 1–3 (frequent) |
|  | b. Speak on the phone with children | Same as above | Recoded: 1 = 4–6, 0 = 1–3 |
|  | c. Write or email children | Same as above | Recoded: 1 = 4–6, 0 = 1–3 |
|  | ***Total scoring rule*** |  | Any contact = 0; if no children, score = 1.  Range: 0–1 |
| **Contact with Other Family Members** | a. Meet up with other family members (arranged or chance meetings) | Same 6-category scale | Recoded: 1 = 4–6, 0 = 1–3 |
|  | b. Speak on the phone with family members | Same scale | Recoded: 1 = 4–6, 0 = 1–3 |
|  | c. Write or email other family members | Same scale | Recoded: 1 = 4–6, 0 = 1–3 |
|  | ***Total scoring rule*** |  | Any contact = 0; if no family, score = 1. Range: 0–1 |
| **Contact with Friends** | a. Meet up with friends (arranged or chance meetings) | Same 6-category scale | Recoded: 1 = 4–6, 0 = 1–3 |
|  | b. Speak on the phone with friends | Same scale | Recoded: 1 = 4–6, 0 = 1–3 |
|  | c. Write or email friends | Same scale | Recoded: 1 = 4–6, 0 = 1–3 |
|  | ***Total scoring rule*** |  | Any contact = 0; if no friends, score = 1.  Range: 0–1 |
| **Composite SI Score^b^** | --- | ---- | Sum of: (1) Living partner (reversed), (2) Contact with children, (3) Contact with other family, (4) Contact with friends. Range: **0–4** 0 = Socially Integrated.  4 = Socially Isolated |

^a^The spouse/partner item was directly included in the scoring, whereas items indicating the presence of children, other family members, and friends were used only to facilitate the calculation of the corresponding contact components^.^

^b^Ertel KA, Glymour MM, Berkman LF. Effects of social integration on preserving memory function in a nationally representative US elderly population. *Am J Public Health*. 2008 Jul;98(7):1215-20. doi: 10.2105/AJPH.2007.113654. Epub 2008 May 29. PMID: 18511736; PMCID: PMC2424091.

**S2 Table 4. Descriptive statistics of the 2022 HRS study sample (n = 615) before multiple imputations.**

|  | **Community of Residence** | | |  | **Place of birth** | | |  | **Region of residence** | | | |
| --- | --- | --- | --- | --- | --- | --- | --- | --- | --- | --- | --- | --- |
| Characteristics | Rual  (n=48) | Urban  (n=566) | *P*-value |  | Foreign-born  (n=395) | US-born  (n=220) | *P*-value |  | Northeast-Midwest  (n=92) | South  (n=242) | West  (n=281) | *P*-value |
| Age—mean(SD) | 68.96 (8.25) | 68.94 (6.94) | 0.99 |  | 69.03 (7.28) | 68.74 (6.62) | 0.62 |  | 69.43 (8.19) | 68.88 (6.79) | 68.8 (6.87) | 0.75 |
| Gender—n(%) |  |  | 0.75 |  |  |  | 0.99 |  |  |  |  | 0.82 |
| Female | 27 (56.2) | 338 (59.7) |  |  | 235 (59.5) | 130 (59.1) |  |  | 52 (56.5) | 146 (60.3) | 167 (59.4) |  |
| Male | 21 (43.8) | 228 (40.3) |  |  | 160 (40.5) | 90 (40.9) |  |  | 40 (43.5) | 96 (39.7) | 114 (40.6) |  |
| Race—n(%) |  |  | 0.38 |  |  |  | 0.75 |  |  |  |  | <0.001 |
| Non-white | 25 (52.1) | 245 (43.3) |  |  | 170 (43) | 101 (45.9) |  |  | 58 (63) | 80 (33.1) | 133 (47.3) |  |
| White/Caucasian | 23 (47.9) | 308 (54.4) |  |  | 213 (53.9) | 118 (53.6) |  |  | 31 (33.7) | 161 (66.5) | 139 (49.5) |  |
| Unknown | 0 (0) | 13 (2.3) |  |  | 12 (3) | 1 (0.5) |  |  | 3 (3.3) | 1 (0.4) | 9 (3.2) |  |
| Educational levels—n(%) |  |  | 0.01 |  |  |  | <0.001 |  |  |  |  | 0.32 |
| Primary | 13 (27.1) | 256 (45.2) |  |  | 228 (57.7) | 42 (19.1) |  |  | 41 (44.6) | 114 (47.1) | 115 (40.9) |  |
| Secondary | 22 (45.8) | 238 (42) |  |  | 123 (31.1) | 137 (62.3) |  |  | 43 (46.7) | 93 (38.4) | 124 (44.1) |  |
| Tertiary | 13 (27.1) | 72 (12.7) |  |  | 44 (11.1) | 41 (18.6) |  |  | 8 (8.7) | 35 (14.5) | 42 (14.9) |  |
| Marital status—n(%) |  |  | 0.40 |  |  |  | 0.07 |  |  |  |  | 0.04 |
| Single/Divorced | 21 (43.8) | 206 (36.4) |  |  | 135 (34.2) | 92 (41.8) |  |  | 45 (48.9) | 83 (34.3) | 99 (35.2) |  |
| Married/Partnered | 27 (56.2) | 359 (63.4) |  |  | 260 (65.8) | 127 (57.7) |  |  | 47 (51.1) | 158 (65.3) | 182 (64.8) |  |
| Unknown | 0 (0) | 1 (0.2) |  |  | 0 (0) | 1 (0.5) |  |  | 0 (0) | 1 (0.4) | 0 (0) |  |
| Household income —n(%) |  |  | 0.94 |  |  |  | 0.004 |  |  |  |  | 0.01 |
| ≤ $17,204 | 17 (35.4) | 188 (33.2) |  |  | 146 (37) | 59 (26.8) |  |  | 36 (39.1) | 94 (38.8) | 75 (26.7) |  |
| $17,204–$40,810.67 | 15 (31.2) | 189 (33.4) |  |  | 135 (34.2) | 70 (31.8) |  |  | 28 (30.4) | 83 (34.3) | 94 (33.5) |  |
| >$40,810.67 | 16 (33.3) | 189 (33.4) |  |  | 114 (28.9) | 91 (41.4) |  |  | 28 (30.4) | 65 (26.9) | 112 (39.9) |  |
| Number of chronic diseases—n(%) |  |  | 0.09 |  |  |  | 0.13 |  |  |  |  | 0.94 |
| None | 12 (25) | 93 (16.4) |  |  | 75 (19) | 30 (13.6) |  |  | 15 (16.3) | 42 (17.4) | 48 (17.1) |  |
| 1 | 17 (35.4) | 158 (27.9) |  |  | 116 (29.4) | 60 (27.3) |  |  | 26 (28.3) | 65 (26.9) | 85 (30.2) |  |
| 2+ | 19 (39.6) | 315 (55.7) |  |  | 204 (51.6) | 130 (59.1) |  |  | 51 (55.4) | 135 (55.8) | 148 (52.7) |  |
| Physical activity—n(%) |  |  | 0.03 |  |  |  | 0.73 |  |  |  |  | 0.24 |
| Physically inactive | 9 (18.8) | 201 (35.5) |  |  | 138 (34.9) | 73 (33.2) |  |  | 32 (34.8) | 92 (38) | 87 (31) |  |
| Physically active | 39 (81.2) | 365 (64.5) |  |  | 257 (65.1) | 147 (66.8) |  |  | 60 (65.2) | 150 (62) | 194 (69) |  |
| Alcohol consumption—n(%) |  |  | 0.22 |  |  |  | 0.04 |  |  |  |  | 0.85 |
| No drinking | 27 (56.2) | 386 (68.2) |  |  | 279 (70.6) | 134 (60.9) |  |  | 65 (70.7) | 164 (67.8) | 184 (65.5) |  |
| Low-to-moderate frequency | 18 (37.5) | 148 (26.1) |  |  | 98 (24.8) | 69 (31.4) |  |  | 21 (22.8) | 65 (26.9) | 81 (28.8) |  |
| Higher-frequency drinking | 3 (6.2) | 32 (5.7) |  |  | 18 (4.6) | 17 (7.7) |  |  | 6 (6.5) | 13 (5.4) | 16 (5.7) |  |
| Social isolation— mean(SD) | 0.94 (0.95) | 0.99 (0.91) | 0.79 |  | 0.96 (0.89) | 1.01 (0.94) | 0.60 |  | 1.11 (1.02) | 1.01 (0.93) | 0.91 (0.86) | 0.29 |
| Unknown | 14 (29.17) | 191 (33.75) |  |  | 152 (38.48) | 54 (24.55) |  |  | 29 (31.52) | 78 (32.23) | 99 (35.23) |  |
| Intrinsic capacity—mean(SD) | 6.92 (1.23) | 6.45 (1.71) | 0.02 |  | 6.52 (1.67) | 6.41 (1.7) | 0.42 |  | 6.45 (1.54) | 6.36 (1.78) | 6.6 (1.63) | 0.25 |

*Notes*: Continuous variables are presented as mean (SD) and compared using independent two-sample t tests for two-group comparisons or one-way analysis of variance (ANOVA) for comparisons across three groups. Categorical variables are presented as n (%) and compared using Pearson’s chi-square tests or Fisher’s exact tests when appropriate. *P-*values were calculated using observed (pre-imputation) data, with missing observations excluded from each analysis.

**S2 Table 5. Comparison of baseline characteristics between included (n = 615) and excluded (n = 1,480) participants in the 2022 HRS wave before multiple imputation.**

|  | **Value** | **Included sample**  **(n=615)** |  | **Excluded sample**  **(n=1,480)** | ***P*-value** |
| --- | --- | --- | --- | --- | --- |
| Age—mean(SD) | Numerical | 68.92(7.04) |  | 70.27(8.13) | **<0.001** |
| Gender—n(%) |  |  |  |  | **0.48** |
|  | Female | 365 (59.35) |  | 852 (57.57) |  |
|  | Male | 250 (40.65) |  | 628 (42.43) |  |
| Race— n(%) |  |  |  |  | **0.14** |
|  | Non-white | 271 (44.07) |  | 601 (40.61) |  |
|  | White/Caucasian | 331 (53.82) |  | 852 (57.57) |  |
|  | Unknown | 13 (2.11) |  | 27 (1.82) |  |
| Educational levels— n(%) |  |  |  |  | **0.60** |
|  | Primary | 270 (43.9) |  | 654 (44.19) |  |
|  | Secondary | 260 (42.28) |  | 599 (40.47) |  |
|  | Tertiary | 85 (13.82) |  | 227 (15.34) |  |
| Marital status— n(%) |  |  |  |  | **0.25** |
|  | Single/Divorced | 227 (36.91) |  | 585 (39.53) |  |
|  | Married/Partnered | 387 (62.93) |  | 886 (59.86) |  |
|  | Unknown | 1 (0.16) |  | 9 (0.61) |  |
| Household income — n(%) |  |  |  |  | **0.59** |
|  | ≤ $17,204 | 205 (33.33) |  | 528 (35.68) |  |
|  | $17,204–$40,810.67 | 205 (33.33) |  | 476 (32.16) |  |
|  | > $40,810.67 | 205 (33.33) |  | 476 (32.16) |  |
| Community of residence — n(%) |  |  |  |  | **<0.001** |
|  | Rural | 48 (7.8) |  | 196 (13.24) |  |
|  | Urban | 566 (92.03) |  | 1254 (84.73) |  |
|  | Unknown | 1 (0.16) |  | 30 (2.03) |  |
| Region of residence— n(%) |  |  |  |  | **0.43** |
|  | Northeast-Midwest | 92 (14.96) |  | 216 (14.59) |  |
|  | South | 242 (39.35) |  | 613 (41.42) |  |
|  | West | 281 (45.69) |  | 621 (41.96) |  |
|  | Unknown |  |  | 30 (2.03) |  |
| Place of birth— n(%) |  |  |  |  | **0.89** |
|  | Foreign-born | 395 (64.23) |  | 943 (63.72) |  |
|  | US-born | 220 (35.77) |  | 535 (36.15) |  |
|  | Unknown |  |  | 2 (0.14) |  |
| Number of chronic diseases—n(%) |  |  |  |  | **0.82** |
|  | None | 105 (17.07) |  | 264 (17.84) |  |
|  | 1 | 176 (28.62) |  | 434 (29.32) |  |
|  | 2+ | 334 (54.31) |  | 782 (52.84) |  |
| Physical activity— n(%) |  |  |  |  | **1.00** |
|  | Physically inactive | 211 (34.31) |  | 507 (34.26) |  |
|  | Physically active | 404 (65.69) |  | 970 (65.54) |  |
|  | Unknown |  |  | 3 (0.2) |  |
| Alcohol consumption— n(%) |  |  |  |  | **0.22** |
|  | No drinking | 413 (67.15) |  | 1015 (68.58) |  |
|  | Low-to-moderate frequency | 167(27.15) |  | 356 (24.05) |  |
|  | Higher-frequency drinking | 35(5.69) |  | 105 (7.09) |  |
|  | Unknown |  |  | 4 (0.27) |  |
| Social isolation—mean(SD) | Numerical | 0.98(0.91) |  | 1.37(1.13) | **0.002** |
|  | Unknown | 206 |  | 1385 |  |
| Intrinsic capacity —mean(SD) | Numerical | 6.48(1.68) |  | 5.38(1.65) | **<0.001** |

*Notes*: All included and excluded participants were Hispanic adults aged ≥60 years. *P*-values were calculated using available cases for each characteristic. Continuous and categorical variables were compared using independent-sample t-tests and Pearson’s chi-square tests, respectively.

**S2 Table 6. Multicollinearity assessment of variables included in the regression model before multiple imputations (n = 615).**

|  | **VIF** | **95% CI** | **Adjusted VIF** | **Tolerance** | **Tolerance 95% CI** |
| --- | --- | --- | --- | --- | --- |
| Age | 1.14 | [1.06, 1.32] | 1.07 | 0.88 | [0.76, 0.95] |
| Social isolation | 1.37 | [1.24, 1.57] | 1.17 | 0.73 | [0.64, 0.81] |
| Gender | 1.15 | [1.07, 1.34] | 1.07 | 0.87 | [0.75, 0.94] |
| Race | 1.11 | [1.04, 1.31] | 1.05 | 0.90 | [0.77, 0.96] |
| Marital status | 1.69 | [1.50, 1.95] | 1.30 | 0.59 | [0.51, 0.67] |
| Education level | 1.52 | [1.36, 1.74] | 1.11 | 0.66 | [0.57, 0.74] |
| Number of chronic diseases | 1.13 | [1.05, 1.32] | 1.03 | 0.88 | [0.76, 0.95] |
| Household income | 1.67 | [1.48, 1.92] | 1.14 | 0.60 | [0.52, 0.67] |
| Alcohol consumption | 1.18 | [1.09, 1.37] | 1.04 | 0.84 | [0.73, 0.92] |
| Physically active | 1.13 | [1.05, 1.32] | 1.06 | 0.88 | [0.76, 0.95] |
| Community of residence | 1.19 | [1.09, 1.37] | 1.09 | 0.84 | [0.73, 0.91] |
| Region of residence | 1.26 | [1.15, 1.45] | 1.06 | 0.79 | [0.69, 0.87] |
| Place of birth | 1.35 | [1.22, 1.55] | 1.16 | 0.74 | [0.65, 0.82] |

^Note: VIF= Variance Inflation Factor; a measure of multicollinearity. Values close to 1 indicate low multicollinearity, while values above 5 or 10 are often considered high. Tolerance: The reciprocal of VIF (1/VIF). Low tolerance values (close to 0) indicate high multicollinearity.^

**S2 Table 7. Pooled multiple linear regression results for intrinsic capacity after multiple imputations in the full sample (n = 615).**

|  | **Overall model without interaction** | **Overall model with interaction among social isolation*region of residence** | **Overall model with interaction among social isolation*place of birth** | **Overall model with interaction among social isolation*** **Community of residence** |
| --- | --- | --- | --- | --- |
|  | **β(SE)** | **β(SE)** | **β(SE)** | **β(SE)** |
| Age group | 0.05*** (0.01) | 0.05*** (0.01) | 0.05***(0.01) | 0.05***(0.01) |
| Social isolation | -0.02 (0.13) | -0.29 (0.18) | -0.02 (0.17) | -0.39* (0.19) |
| Region of residence |  |  |  |  |
| Northeast-Midwest(ref.) |  |  |  |  |
| South | -0.14 (0.24) | -0.47 (0.41) | -0.14 (0.25) | -0.15 (0.24) |
| West | -0.26 (0.24) | -0.71* (0.34) | -0.26 (0.24) | -0.27 (0.24) |
| Social isolation* Region of residence |  |  |  |  |
| socisol:region Northeast-Midwest(ref.) |  |  |  |  |
| socisol:regionSouth |  | 0.26 (0.25) |  |  |
| socisol:regionWest |  | 0.39 (0.24) |  |  |
| Gender |  |  |  |  |
| Female(ref.) |  |  |  |  |
| Male | -0.08 (0.22) | -0.09 (0.22) | -0.08 (0.23) | -0.07 (0.22) |
| Race |  |  |  |  |
| Non-white(ref.) |  |  |  |  |
| White/Caucasian | -0.09 (0.19) | -0.09 (0.19) | -0.08 (0.19) | -0.07 (0.19) |
| Educational levels |  |  |  |  |
| Primary (ref.) |  |  |  |  |
| Secondary | 0.07 (0.22) | 0.06 (0.22) | 0.07 (0.22) | 0.09 (0.22) |
| Tertiary | 0.66 (0.42) | 0.65 (0.42) | 0.67 (0.43) | 0.66 (0.42) |
| Marital status |  |  |  |  |
| Single/Divorced (ref.) |  |  |  |  |
| Married/Partnered | 0.35 (0.23) | 0.35 (0.23) | 0.35 (0.23) | 0.36 (0.23) |
| Household income |  |  |  |  |
| ≤ $17,204(ref.) |  |  |  |  |
| $17,204–$40,810.67 | 0.57* (0.26) | 0.56* (0.26) | 0.57* (0.25) | 0.55* (0.26) |
| > $40,810.67 | 0.69**(0.26) | 0.70** (0.25) | 0.69** (0.26) | 0.68* (0.26) |
| Community of residence |  |  |  |  |
| Rural (ref.) |  |  |  |  |
| Urban | -0.29 (0.19) | -0.31 (0.21) | -0.28 (0.19) | -0.68* (0.28) |
| Social isolation*Community of residence |  |  |  |  |
| socisol:residence_urbanruralRural (ref.) |  |  |  |  |
| socisol:residence_urbanruralUrban |  |  |  | 0.40 (0.23) |
| Place of birth |  |  |  |  |
| Foreign-born(ref.) |  |  |  |  |
| US-born | -0.04 (0.20) | -0.05 (0.20) | -0.04 (0.28) | -0.04 (0.20) |
| Social isolation*Place of birth |  |  |  |  |
| socisol:birthplaceForeign-born(ref.) |  |  |  |  |
| socisol:birthplaceUS-born |  |  | -0.00 (0.21) |  |
| Number of chronic diseases |  |  |  |  |
| None(ref.) |  |  |  |  |
| 1 | -0.19 (0.21) | -0.17 (0.21) | -0.19 (0.23) | -0.19 (0.21) |
| ≥ 2 | -1.13*** (0.19) | -1.13*** (0.19) | -1.13*** (0.20) | -1.15*** (0.19) |
| Physical activity |  |  |  |  |
| Physically inactive (ref.) |  |  |  |  |
| Physically active | 0.17 (0.23) | 0.17 (0.23) | 0.17 (0.24) | 0.18 (0.23) |
| Alcohol consumption |  |  |  |  |
| No drinking (ref.) |  |  |  |  |
| Low-to-moderate frequency | 0.20 (0.15) | 0.20 (0.15) | 0.20 (0.15) | 0.19 (0.15) |
| Higher-frequency drinking | 0.10 (0.48) | 0.09 (0.48) | 0.10 (0.49) | 0.12 (0.49) |

*Notes*: Estimates were derived from survey-weighted regression models and pooled across 30 multiply imputed datasets using Rubin’s rules. SE = standard error; **p* < .05; ***p* < .01; ****p* < .001.
